# Combined RIS and EBG Surfaces Inspired Meta-Wearable Textile MIMO Antenna Using Viscose-Wool Felt

**DOI:** 10.3390/polym14101989

**Published:** 2022-05-13

**Authors:** Amira Nur Suraya Shamsuri Agus, Thennarasan Sabapathy, Muzammil Jusoh, Mahmoud A. Abdelghany, Kabir Hossain, Surentiran Padmanathan, Samir Salem Al-Bawri, Ping Jack Soh

**Affiliations:** 1 Advanced Communication Engineering (ACE), Centre of Excellence, Universiti Malaysia Perlis (UniMAP), Jalan Tiga, Pengkalan Jaya Business Centre, Kangar 01000, Malaysia; miraanssa1991@gmail.com (A.N.S.S.A.); suren_wgen@hotmail.com (S.P.); 2 Faculty of Electronic Engineering Technology, Universiti Malaysia Perlis (UniMAP), Kampus Alam UniMAP Pauh Putra, Arau 02600, Malaysia; 3 Department of General Educational Development, Faculty of Science and Information Technology (FSIT), Daffodil International University, Dhaka 1207, Bangladesh; 4 Electrical Engineering Department, College of Engineering, Prince Sattam Bin Abdulaziz University, Wadi Addwasir 11991, Saudi Arabia; 5 Department of Electrical Engineering, Faculty of Engineering, Minia University, Minia 61519, Egypt; abdelghany@mu.edu.eg; 6 Faculty of Engineering, Norwegian University of Science and Technology (NTNU), N-2815 Gjøvik, Norway; kabir.hossain@ntnu.no; 7 Space Science Centre, Climate Change Institute, Universiti Kebangsaan Malaysia, Bangi 43600, Malaysia; s.albawri@gmail.com; 8 Centre for Wireless Communications (CWC), University of Oulu, 90014 Oulu, Finland; pingjack.soh@oulu.fi

**Keywords:** metasurface, metamaterials, high performance textiles, wearable antenna, textile antennas, polymer

## Abstract

In this paper, we present a textile multiple-input–multiple-output (MIMO) antenna designed with a metamaterial inspired reactive impedance surface (RIS) and electromagnetic bandgap (EBG) using viscose-wool felt. Rectangular RIS was used as a reflector to improve the antenna gain and bandwidth to address well known crucial challenges—maintaining gain while reducing mutual coupling in MIMO antennas. The RIS unit cell was designed to achieve inductive impedance at the center frequency of 2.45 GHz with a reflection phase of 177.6°. The improved bandwidth of 170 MHz was achieved by using a square shaped RIS under a rectangular patch antenna, and this also helped to attain an additional gain of 1.29 dBi. When the antenna was implemented as MIMO, a split ring resonator backed by strip line type EBG was used to minimize the mutual coupling between the antenna elements. The EBG offered a sufficient band gap region from 2.37 GHz to 2.63 GHz. Prior to fabrication, bending analysis was carried out to validate the performance of the reflection coefficient (*S*_11_) and transmission coefficient (*S*_21_). The results of the analysis show that bending conditions have very little impact on antenna performance in terms of S-parameters. The effect of strip line supported SRR-based EBG was further analyzed with the fabricated prototype to clearly show the advantage of the designed EBG towards the mutual coupling reduction. The designed MIMO-RIS-EBG array-based antenna revealed an *S*_21_ reduction of −9.8 dB at 2.45 GHz frequency with overall *S*_21_ of <−40 dB. The results also indicated that the proposed SRR-EBG minimized the mutual coupling while keeping the mean effective gain (MEG) variations of <3 dB at the desired operating band. The specific absorption rate (SAR) analysis showed that the proposed design is not harmful to human body as the values are less than the regulated SAR. Overall, the findings in this study indicate the potential of the proposed MIMO antenna for microwave applications in a wearable format.

## 1. Introduction

Antenna design for on-body applications has been popular in the past few decades. On-body antennas are mainly known for wireless body area networks (WBAN) and they are designed for various applications such as emergency rescue services, global positioning systems (GPS) [1] and health monitoring [2]. The antenna’s performance for the WBAN antenna is crucial because the antenna is placed close to the human body. As a result, some considerations are usually given to antennas for on-body applications where, (1) deformation analysis is usually carried out to check the performance of the antenna under various bending conditions (if the antenna is designed using flexible material), (2) specific absorption rate analysis is measured for the antenna with on-body application to verify the safety of the antenna’s electromagnetic (EM) effect on the human body and (3) antenna characteristics such as *S*_11_ and gain analysis with the on-body condition.

The choice of material becomes essential whenever an antenna is designed using flexible materials. Using polymer as a flexible material has been a common practice in wearable antenna designs [3,4]. The comprehensive review conducted in [4] has revealed that EM radiation characteristics of the antennas are affected hugely when the flexible polymer-based antennas undergo bending. In general, antenna designs consist of dielectric material as the substrate and conductive material as the patch that serves as the radiating element. Mixed metals with fabrics and conductive inks are some examples of conductive materials that have been adopted in previous works [5]. Polymers are widely been used as conductive materials in antenna design. They have been used as conductive threads [6], conductive polymers [7] and conductive textiles [8]. In addition, polymers are also commonly used as dielectric material or substrates used in antenna design. In [9], the substrate of the antenna has adopted the use of viscose-wool felt since it provides easier fabrication with sufficient flexibility while enabling strong adhesion with conductive textile Shieldit Super^TM^. Apart from antenna design, polymers are also widely used in applications such as energy harvesting [10], supercapacitor [11,12,13], tissue engineering [14], immunosensor [15] and gas sensor [16]. As for the on-body application, flexible wideband antennas based on polymer technology have been proposed for medical imaging systems [17,18]. 

Recent research has shown that multiple-input–multiple-output (MIMO) antennas were also designed for WBAN applications to overcome the multipath fading that can happen due to the on-body communication links [19,20,21]. Reflections or scatterings that occur around the human body or the surrounding environment cause multipath fading. As a result, the reliability of multi-signal communication, and the performance of a WBAN system are reduced [22]. A diversity technique such as MIMO is needed to improve effective communication under the influence of multipath fading. Therefore, designing MIMO antenna with lower mutual coupling between antenna elements becomes significant to overcome the multipath fading issue. In the last decade, a few works that designed MIMO wearable antennas have been reported. In [23], a dumbbell-shaped stub on the ground was used for a wearable MIMO antenna to reduce mutual coupling. For wearable 5G devices, a folded-dipole MIMO antenna was developed in [20]. Likewise, an ultra-wide-band (UWB) MIMO antenna was designed for wearable devices with C-shaped slots to improve isolation [21]. Although these works have designed MIMO, the antennas designed are not directly used for on-body applications and the material used is not flexible. The design and analysis of the MIMO wearable antenna can be found in [24]. In that work, reported important results for on-body condition such as bending and specific absorption rate (SAR). Although the design outperforms other related works in terms of isolation with a limited gap between elements, the MIMO was not implemented with a common ground. MIMO designs need to have a single ground plane for MIMO to ensure the system has a common reference level (zero for ground) thus all the signals in the system can be interpreted properly [25].

The use of metamaterials or metasurfaces for on-body antenna design has found its interest to attain various performance improvements. Antenna characteristics such as gain, bandwidth and directivity are usually improved with the use of metamaterial. For instance, the artificial magnetic conductor (AMC) is used to improve gain and improve bandwidth [1,26]. In [27], a via-less EBG was designed for wearable antenna to increase the antenna gain and front-to-back ratio (FBR). In particular, minimal works have adopted metamaterial to solve the mutual coupling issue in flexible/textile MIMO antennas. A recently reported work [28] has adopted electromagnetic bandgap (EBG) to improve the isolation between dual-band MIMO antennas. However, this work lacks analyses such as deformation and SAR examination. 

This research work presents a wearable textile MIMO antenna featuring two types of metasurface. First, a reactive impedance surface (RIS) array was designed to improve the antenna bandwidth and gain. Then, a split ring resonator (SRR) backed with strip line based EBG was implemented to reduce the mutual coupling between the multiple antennas. To the best of the authors’ knowledge, the use of different types of metasurface in a MIMO wearable antenna has never been investigated, especially when flexible materials are used. The metasurfaces and the antenna were designed using viscose-wool felt. The following sections present a comprehensive insight into the proposed work’s design stages.

## 2. Flexible Polymer-Based Meta-Wearable Antenna Design 

Polymer-based flexible material was adopted for the proposed meta-wearable antenna. Flexible polymers have been studied in many recently investigated antenna research works [4]. The three main components of this work, namely RIS, EBG and the antenna, were all designed using a flexible polymer. Shieldit Super^TM^ with a thickness of 0.17 mm and a conductivity value of 1.18 × 10^5^ S/m was used as the ground plane of the RIS structure, EBG structure and the radiator. The commercially available Shieldit Super^TM^ is made from a rugged rip-stop polyester substrate, conductive nickel and copper plating. The other side of the sheet is coated with a non-conductive hot melt adhesive. This ensures the sheet is easily ironed onto the textile substrates. Meanwhile, a viscose-wool felt with a thickness of 3 mm, a dielectric constant of 1.44 and a loss tangent of 0.044 was employed as the substrate. Existing EM related works using this felt with Shiedlit Super^TM^ have shown good performance where the simulated and measured results were approximately the same [4,9,18]. The felt consists of 70% wool and 30% viscose which forms a good composition of fibers with a density of 0.25 gm/CC. This property ensured the Shieldit Super^TM^ can be easily ironed and attached to the viscose-wool felt. Apart from that, it meets British Standard 4060 for pressed wool felts for reliability and quality tests thus it was adopted as the substrate for the metasurfaces and antenna design. Computer Simulation Technology (CST)-Microwave Studio Suite (MWS) was used to model and simulate the metasurfaces and meta-inspired antenna. The analyses of these structures are reported in the following subsections.

### 2.1. Reactive Impedance Surface Design with Rectangular Patch Antenna

**Figure 1:**
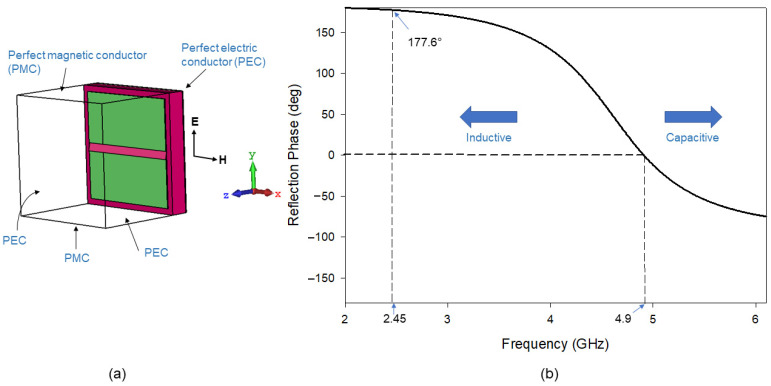
RIS unit cell simulation: (**a**) RIS unit cell model and (**b**) reflection phase diagram.

In this work, a square-shaped RIS unit cell [29,30] with a dimension of *a* × *a* was modeled and simulated. The square shape was adopted due to its simplicity in design and fabrication of the antenna using flexible materials. The optimized dimension values, *a* is 18 mm and the gap between unit cell, *g* is 3 mm. As shown in Figure 1a, the unit cell RIS was backed by a perfect electric conductor (PEC) and interacted with transverse electromagnetic wave (TEM) from +*z* direction, establishing PEC and perfect magnetic conductor (PMC) boundaries perpendicular to the incident electric (E) and magnetic (H) fields. The resonant frequency of the RIS substrate is *f_RIS_* = 4.9 GHz, at which point the substrate acts like a PMC (open circuit). The RIS acts as an inductor below this resonance frequency. In particular, as shown in Figure 1b, at 2.45 GHz, the RIS acts as an inductor with a reflection phase at 177.6°. At this range, the surface can store magnetic energy, and this magnetic energy will compensate for the electric energy associated with a patch antenna. 

**Figure 2:**
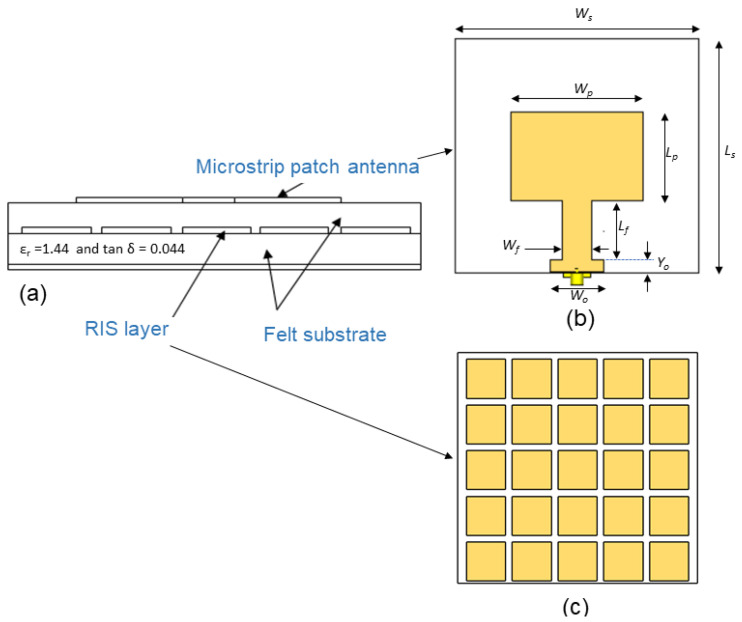
Schematic diagram of the proposed antenna and the RIS array: (**a**) side view of patch antenna with RIS, (**b**) First layer: front view of patch antenna and (**c**) Second layer: 5 × 5 array of RIS.

Figure 2 presents a structure of a grounded dual-layer substrate with similar relative permittivity and height. We adopted a rectangular patch antenna in this design. At the same time, the proposed RIS metasurface was modeled as an array on the top of the lower layer, i.e., at the interface between both substrates. 

The coaxial cable was connected at the edge of the line, whose width and length were set to match the antenna at 50 Ohm. These two parameters and the patch length were optimized to increase the gain, widen the bandwidth and miniaturize the antenna size at the frequency of 2.45 GHz. We optimized the patch antenna and RIS dimensions to attain the best performance of the MIMO antenna design which describes in the next section. Table 1 lists the optimized dimensions of the patch antenna.

### 2.2. Electromagnetic Band-Gap Design

**Figure 3:**
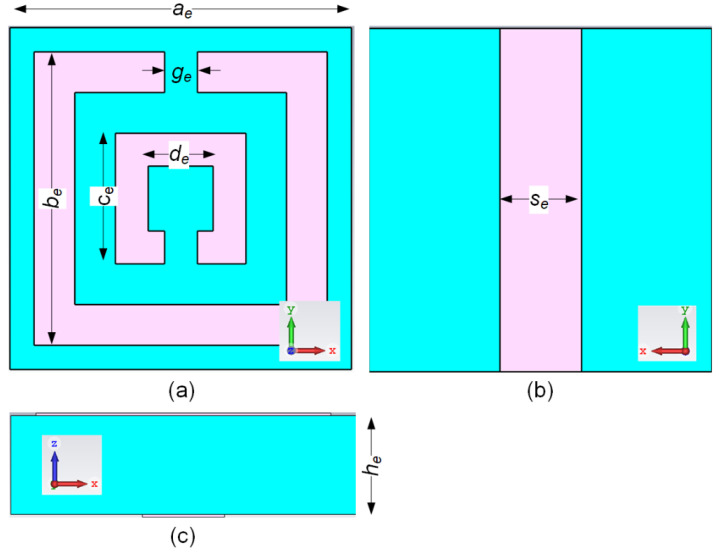
EBG unit cell structure: (**a**) top view, (**b**) bottom view and (**c**) side view.

The EBG unit cell simulation was conducted using the Eigenmode Solver in CST MWS. The dispersion diagram method recommended in [31] was used to examine the properties of the EBG unit cell. We chose the SRR structure as it is via-less, thus making it easier for fabrication and integration with viscose-wool felt. The EBG structure with vias may increase the fabrication complexity when using textile-based materials. However, a via-less EBG without any splits on the structure could increase the frequency of mode I [27]. Therefore, the SRR-based EBG was implemented to maintain mode I at a lower frequency and mode II at a higher frequency to obtain a sufficient stop band. Figure 3 shows the unit cell structure of the EBG. The two split rings modelled in the SRR structure are capable of controlling mode I and mode II of the EBG. The parameters of the designed EBG are shown in Table 2. 

**Figure 4:**
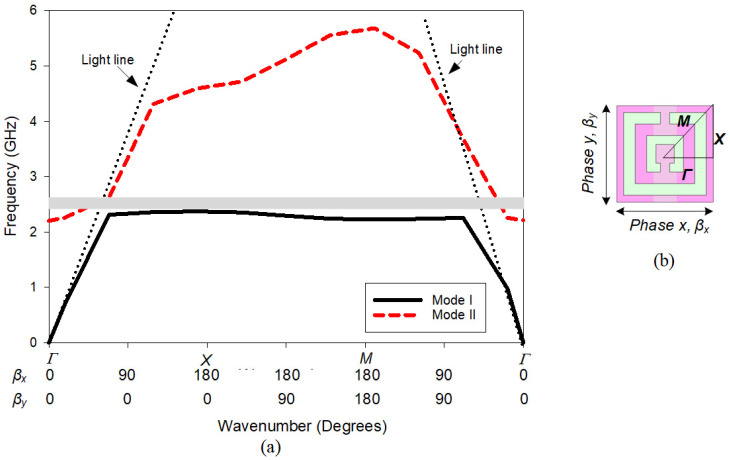
EBG unit cell simulation and result (**a**) Dispersion diagram of SRR backed strip line EBG, (**b**) Brillouin Triangle simulation using Eigen mode solver.

The EBG unit cell simulation was essential to ensure the desired stop band or bandgap region is suitable for the developed MIMO antenna. Figure 4 shows the dispersion diagram of the Brillouin Triangle (Γ-X-M) [27] that corresponds to the Eigenmode simulation of the EBG unit cell shown in Figure 3. Mode I and II are the fundamental modes of transverse magnetic (TM) and the higher mode of transverse electric (TE) polarized waves, respectively. The black dotted lines represent the light lines (no dispersion case). EBG characteristic is obtained between mode I and mode II under the graph area of light-lines. From Figure 4 it can be seen that the bandgap region obtained is from 2.37 GHz to 2.63 GHz. This bandgap is sufficient for the operating frequency of the MIMO antenna, where the operating frequency range is from 2.4 GHz to 2.5 GHz. The EBG characteristic was expected to reduce the mutual coupling of the MIMO antenna result obtained in the previous section. In other words, the proposed EBG could reduce the *S*_12_ or *S*_21_ magnitude.

### 2.3. MIMO Antenna Design Geometry and Configurations

**Figure 5:**
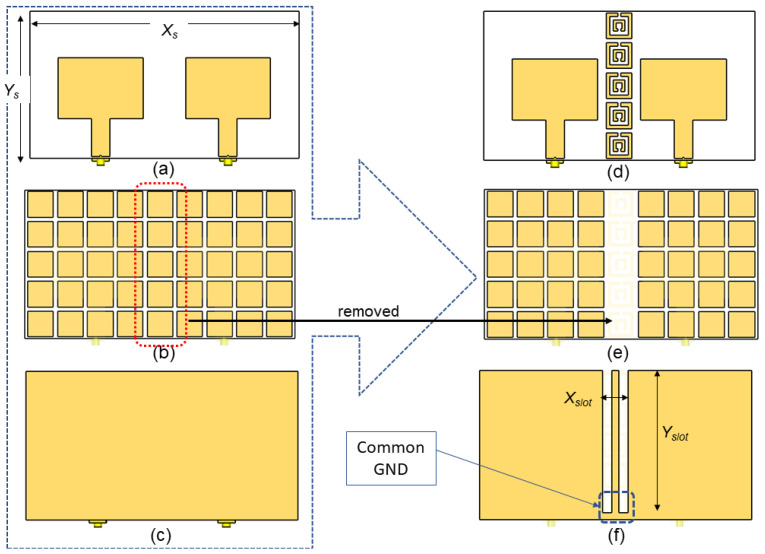
MIMO antenna using RIS: (**a**) front view, (**b**) second layer and (**c**) ground plane; and MIMO antenna using RIS and EBG: (**d**) front view, (**e**) second layer and (**f**) ground plane.

The MIMO-RIS-EBG antenna design flow is illustrated in Figure 5. Since the MIMO antenna consists of two antenna elements, the overall antenna size was increased to *X_s_* × *Y_s_* where *X_s_* = 190 mm and *Y_s_* = 104 mm. Apart from this, *L_p_* was fine-tuned to 43 mm to obtain optimum performance in terms of *S*_11_ when the antenna works as MIMO. To enable strip line for the EBG, a slot was created on the ground plane with a size of *X_slot_* × *Y_slot_* where *X_slot_* = 18 mm and *Y_slot_* = 99 mm. The EBG array was placed between the antenna elements as shown in Figure 4. To accommodate the EBG array, the RIS array that overlaps with the EBG substrate was removed, as depicted in Figure 4e. Although the bottom layer of EBG has strip line, this should not create a direct split on the antenna’s ground plane. Therefore, a common ground plane was ensured at the edge of the strip line as shown in Figure 4f. This is because in a real system the signal should have a single/common ground (GND). Certainly, a direct split can improve the isolation of the antenna element, but this is not a recommended practice by [25].

**Figure 6:**
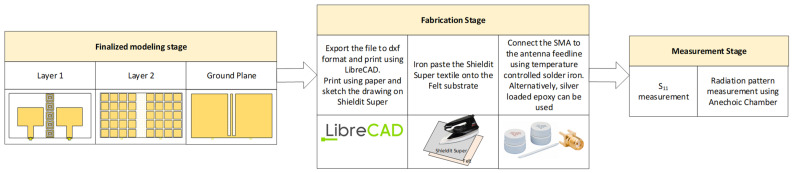
Fabrication process of the meta-wearable textile antenna in this study.

**Figure 7:**
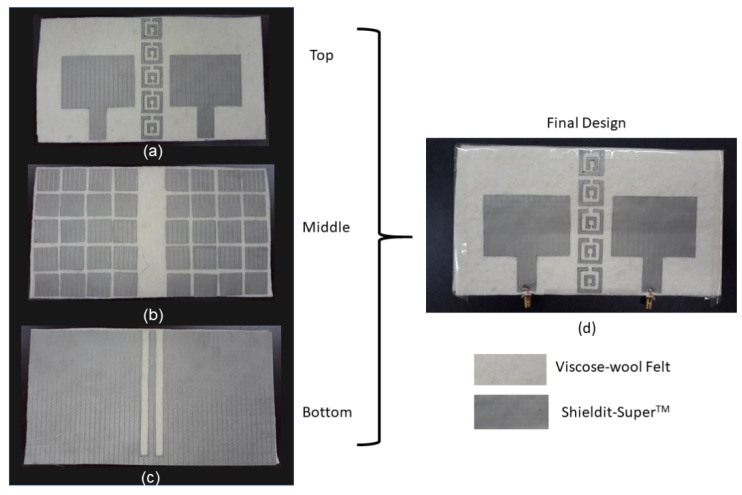
Fabrication MIMO-RIS-EBG antenna using viscose-wool felt and Shieldit-super^TM^: (**a**) front view of the antenna with substrate 1, (**b**) middle view of the antenna with substrate 2 and (**c**) bottom view of the antenna and (**d**) final antenna design.

The steps were used to fabricate the prototype as shown in Figure 6. These steps were adopted from the literature that used similar polymer and conductive materials [9,32]. After finalizing the modeling in the simulation, the structures were printed using computer aided design (CAD) software. The dielectric polymer material (viscose-wool felt) and conductive material (Shieldit Super^TM^) of the prototype were then cut. An iron with medium heat was then used to paste the Shieldit Super^TM^ on the viscose-wool felt. Alternatively, the Shieldit SuperTM can be sewn to the viscose-wool felt. This could ensure the bonding between them remain strong even after washing or repeated bending. The final fabricated prototype is shown in Figure 7. The figure shows clearly the two layers of the antenna: the top layer with a patch antenna and EBG array (no patch is underneath this layer); the middle layer that consists of the RIS array; and the bottom layer that consists of the strip line that is associated with the EBG array of the top layer.

## 3. Results and Discussion

This section presents the related results at each stage of the design. First, the simulated results in terms of *S*_11_ and gain are presented for the single element patch antenna developed with the RIS. The rest of the section discusses the MIMO-RIS-EBG antenna results in various terms such as S-parameter, gain, radiation pattern and mutual coupling analysis.

### 3.1. Advantages of RIS for Patch Antenna

**Figure 8:**
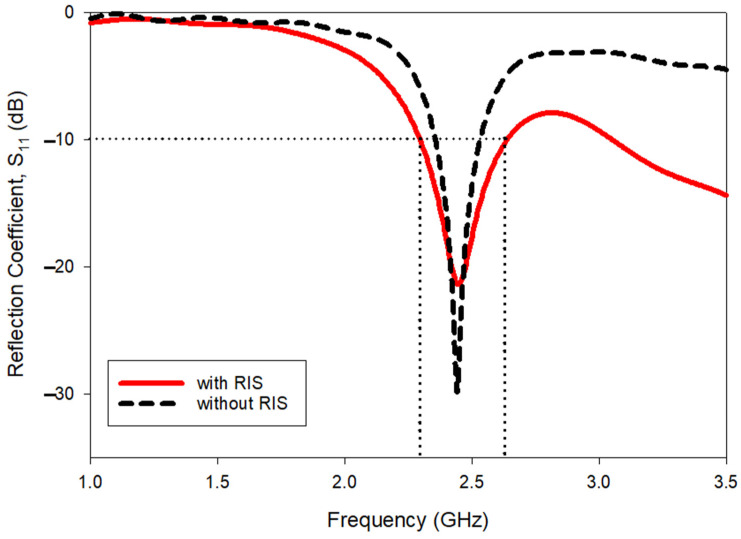
Reflection coefficient result comparison for MPA without RIS and with RIS.

From Figure 8, it is evident that the *S*_11_ bandwidth of a single patch antenna with RIS is 2.292 GHz–2.632 GHz (340 MHz). Meanwhile, the operating bandwidth without RIS is 2.354 GHz–2.535 GHz (181 MHz). Additional bandwidth of almost 170 MHz is obtained with the RIS. Therefore, it is evident that the use of RIS gives more significant greater benefits in terms of bandwidth enhancement.

Apart from this, the RIS also gives advantages in terms of size reduction. Without RIS, the length of the antenna *L*_p_ was 48 mm. With the RIS, the *L*_p_ could be reduced to 39.5 mm. Approximately 18% of the size reduction was attained with the use of RIS.

**Figure 9:**
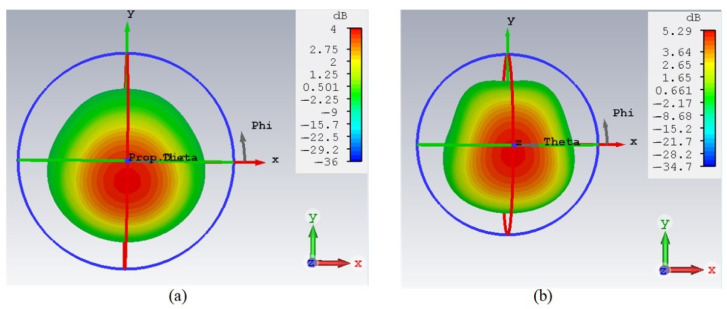
3D radiation pattern results (**a**) patch antenna without RIS (**b**) patch antenna with RIS.

The radiation pattern is another important result that was investigated to see the advantages of using RIS. Figure 9 shows that the gain of the antenna with RIS is greater than the gain of the antenna without RIS. The attainable antenna gain without RIS is 4 dB, while the inclusive RIS layer provided an improved gain of 5.29 dB. This attribute was mainly contributed by the RIS layer when it acts as an inductor at 2.45 GHz. Meanwhile, the RIS surface stored magnetic energy, and this magnetic energy compensated for the electric energy associated with the patch antenna. This helped the EM radiation be further reflected toward the +*z* direction while the back-lobe radiation was reduced. Overall, an additional gain of 1.29 dB could be obtained with the use of RIS. It can be noted that the use of RIS not only improved the bandwidth and reduced the size, but also increased the antenna gain.

### 3.2. Performance Enhancement by Stripline Backed SRR-EBG

**Figure 10:**
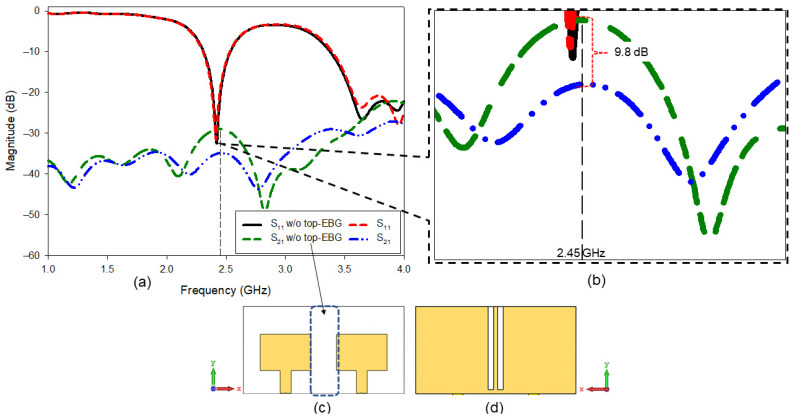
Investigation on the effect of strip line (without EBG top layer) at the back of the antenna: (**a**) S-parameter results, (**b**) enlarged view of the *S_12_* results at 2.45 GHz, (**c**) front view of the antenna without EBG top layer and (**d**) back view of the antenna without EBG top layer.

S-parameter results were then investigated for the final design (MIMO-RIS-EBG). Motivated by previous work, EBG is capable of reducing mutual coupling between the antenna [33]. In this work, we designed and analyzed a new EBG structure to deploy in a RIS based MIMO antenna. Therefore, it was necessary to investigate the effect of EBG cautiously. Additionally, the bottom part of EBG consists of a strip line, thus a careful analysis was carried out to indicate the effect of EBG solely on the performance enhancement in terms of mutual coupling reduction. Figure 10 shows a thorough study directed to see the difference of *S*_21_ result for two conditions as follows, 

Condition 1: simulation result of MIMO with the top part of EBG (as shown in Figure 5c) was removed.

Condition 2: simulation result of MIMO with full part of the EBG, top and bottom part available.

The results in Figure 10 clearly show that the full model of the EBG with the stripline outperforms the antenna without the top EBG structure but with the stripline at the bottom. Without this analysis, one can claim that the *S*_21_ reduction may be due to the defected ground structure formed by the stripline. Approximately the use of SRR EBG backed by strip line reduced the *S*_21_ magnitude by 9.8 dB. Therefore, this investigation provides clear evidence of the EBG performance.

**Figure 11:**
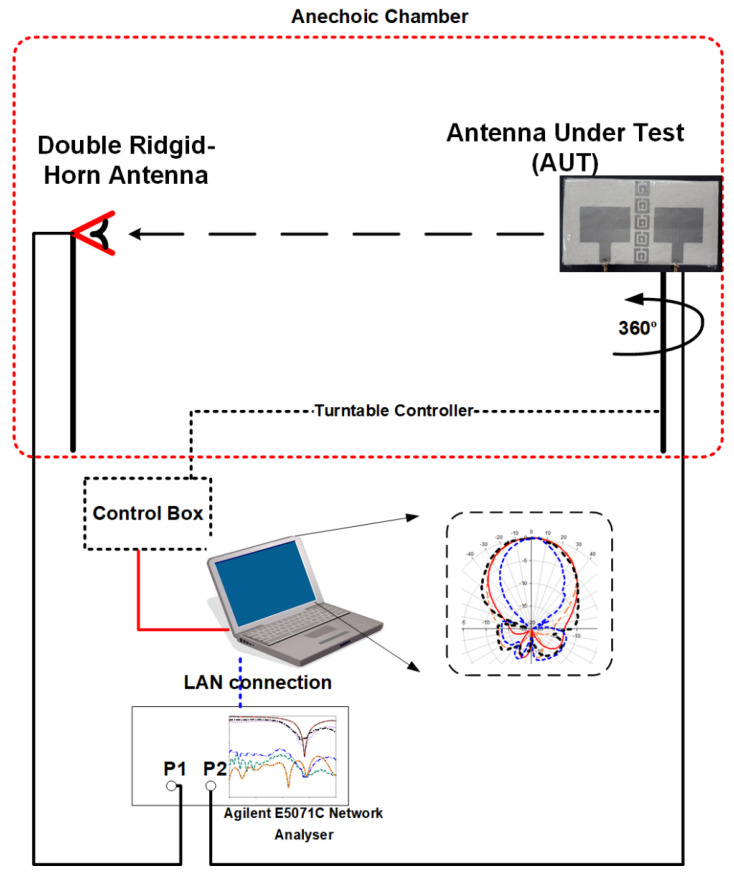
Experimental setup to measure the proposed antenna performance.

The measurements were conducted using the Agilent E5071C Network Analyzer (Agilent Technologies, Bayan Lepas, Penang, Malaysia) to validate the performance of the antenna. Figure 11 illustrates the experimental setup to measure the proposed antenna. To measure the *S*-parameters of the antenna, the coaxial probes from the antenna were connected to port 1 (P1) and port 2 (P2) of the network analyzer. The radiation pattern measurement was conducted with the aid of an Anechoic Chamber and a commercialized double-ridged horn antenna. The P2 of the network analyzer was connected to the antenna under test (AUT) which acts as the receiver. The double-ridged horn antenna (transmitter) was connected to P2. The data from the network analyzer were transferred to the computer using General Purpose Interface Bus (GPIB) cable. 

**Figure 12:**
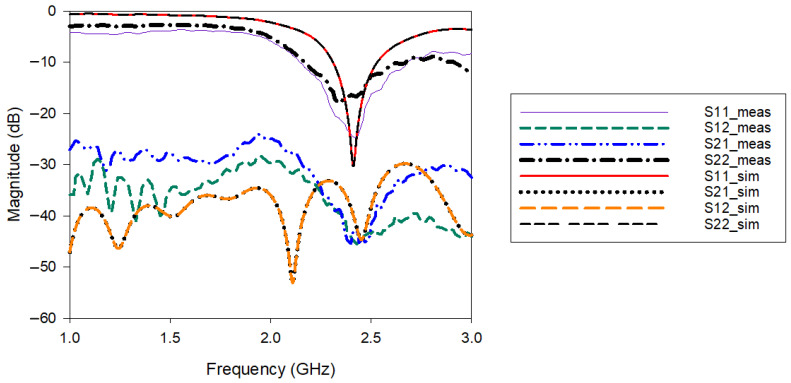
Simulated and measured S-parameters of the MIMO-RIS-EBG.

Figure 12 shows the complete S-parameter results for both simulation and measurement. The simulated and measured *S_11_* indicates a good agreement. The fabricated antenna resonant frequency is slightly shifted both in terms of *S*_11_ and *S*_22_. However, in terms of bandwidth both MIMO elements can cover the wireless body area network and Wi-Fi bandwidth. Apart from that, the measured *S*_21_ and *S*_12_ result indicating the performance of the antenna in terms of mutual coupling reduction also shows reasonable agreement with the simulated results. The measured *S*_11_ bandwidth is from 2.16 GHz to 2.66 GHz. Meanwhile, the measured *S*_21_ and *S*_12_ magnitudes are less than −40 dB at the frequency range from 2.36 to 2.52 GHz.

**Figure 13:**
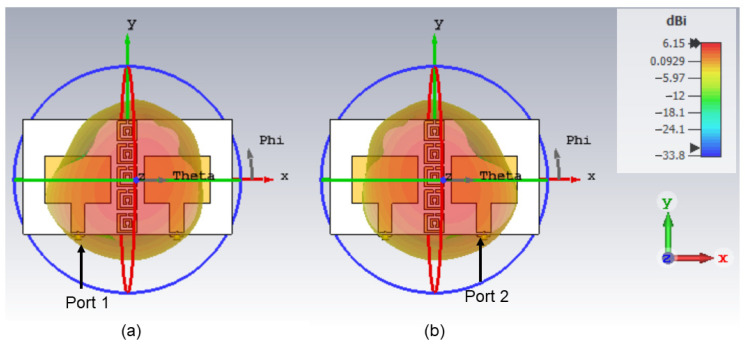
The 3D radiation pattern results of MIMO-RIS-EBG (**a**) antenna 1 and (**b**) antenna 2.

The 3D radiation pattern results shown in Figure 13 indicate that the EBG provides sufficient mutual coupling reduction to ensure the antenna gain is not affected. First, the single antenna gain was improved using RIS from 4 to 5.29 dB as shown in Figure 9. With the implementation of MIMO-RIS, the attainable antenna gain was 5.93 dB. Interestingly, the mutual coupling was further reduced using the proposed EBG, thus finally the MIMO-RIS-EBG antenna achieved 6.15 dB. 

**Figure 14:**
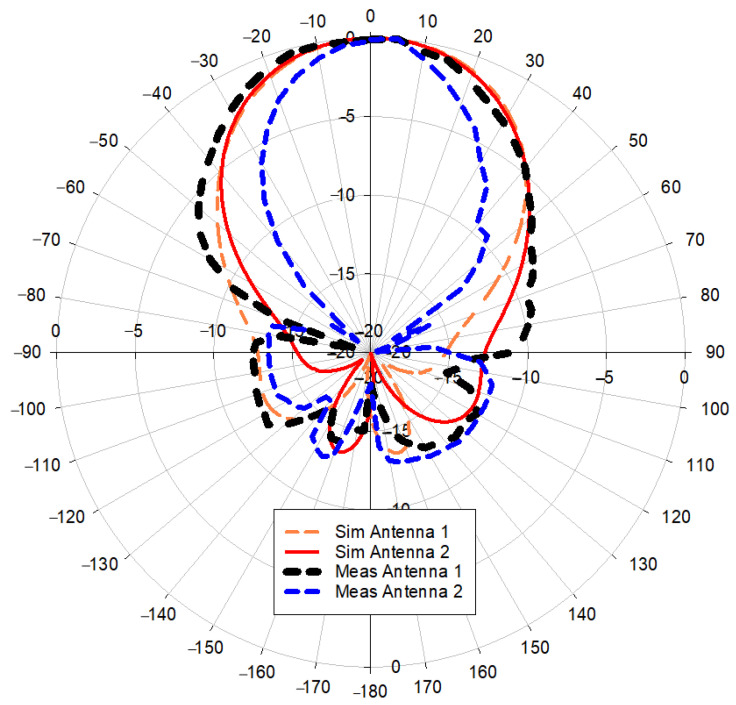
The polar radiation pattern results for MIMO-RIS-EBG.

Figure 14 shows simulated and measured polar radiation pattern results for the MIMO-RIS-EBG antenna. The comparison with simulated results shows that the radiation pattern beamwidth is slightly affected for the antenna at port 2. The other antenna results exhibit a good agreement with the simulated results. The measured gain was also approximately 5.8 dB for antenna elements at both ports.

### 3.3. MIMO Properties of the Proposed Antenna

The performance of the proposed MIMO antenna was carried out in various terms, such as *S*_11_, *S*_21_ and radiation pattern. Additionally, the envelope correlation coefficient (ECC) and mean effective gain (MEG) properties of the antenna were also investigated [34,35]. ECC is a measure of how closely the antenna elements are coupled to each other, and it was calculated using the far-field radiation patterns using Equation (1). The ECC is given by,

(1) $$\mathrm{ECC}=\frac{{\left|\int^{\;}\int_{4\pi }\left(\vec{M_{i}}\left(\theta ,\phi \right)\right)\times \left(\vec{M_{j}}\left(\theta ,\phi \right)\right)d\Omega \right|}^{2}}{\int^{\;}\int_{4\pi }{\left|\left(\vec{M_{i}}\left(\theta ,\phi \right)\right)\right|}^{2}d\Omega \int^{\;}\int_{4\pi }{\left|\left(\vec{M_{j}}\left(\theta ,\phi \right)\right)\right|}^{2}d\Omega }$$

where, *M_i_* and *M_j_* represent the antenna elements, ϕ represents the azimuth angle (0–360 degrees), θ represents elevation angle that pointed by the vector itself, $\vec{M_{i}}\left(\theta ,\phi \right)$ describes the far-field radiation pattern when element/antenna *i* is excited and $\vec{M_{j}}\left(\theta ,\phi \right)$ describes the 3D radiation pattern when element/antenna *j* is excited. Ω represents the solid angle. The acceptable value for ECC is <0.3 [36].

In addition to the ECC, the MEG ratios |MEG*_i_*/MEG*_j_*|, where *i* and *j* denote specific antenna elements that were computed to quantify the imbalanced levels of the diverse propagation branches [37]. The MEG is given by expression (2) where it was assumed that the channel is uniform Rayleigh with equal vertical and horizontal polarization power densities [36]. In other words, MEG then is equal to half of the radiation efficiency.

(2) $$MEG_{i}=0.5\eta_{i,rad}=0.5\left[1-\sum_{j=1}^{M}{\left|S_{ij}\right|}^{2}\right]$$

where *η_i,rad_* is the radiation efficiency, *M* represents total antenna elements and $\left|S_{ij}\right|$ denotes the related scattering parameters.

(3) $$K=\left|MEG_{1}-MEG_{2}\right|<3\;dB$$

where *K* is the MEG variations and must be below 3 dB to have a comparable MEGs.

**Figure 15:**
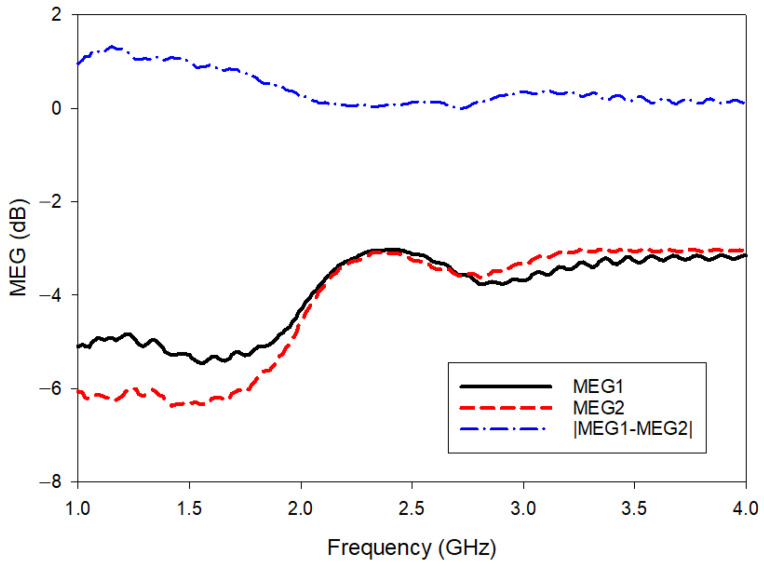
MEG results for MIMO-RIS-EBG.

Figure 15 shows the MEG results of the proposed antenna. It can be noticed that the maximum MEG variation is at a 1.1 GHz frequency with 1.4 dB. At the desired operating range (2.4 to 2.5 GHz), the MEG variation is less than 3 dB. With this, good power balance and low diversity loss can be guaranteed.

**Figure 16:**
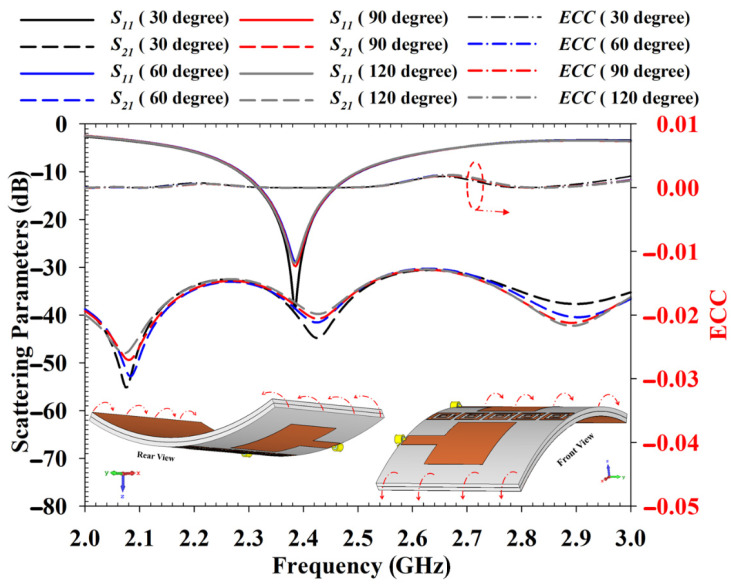
Bending analysis of the antenna at *x* axis.

The performance of the proposed antenna was also validated under deformation analysis. The deformation analysis was conducted with bending conditions applied to the antenna along the *x* and *y* axes. Figure 16 displays the bending analysis carried out on the *x* axis from 30 degrees to 120 degrees. It can be noted that the bending does not affect the antenna results critically. The changes on *S*_11_, *S*_21_ and ECC results are very small due to the bending condition, thus the antenna performance is expected not to be affected severely for on-body application.

**Figure 17:**
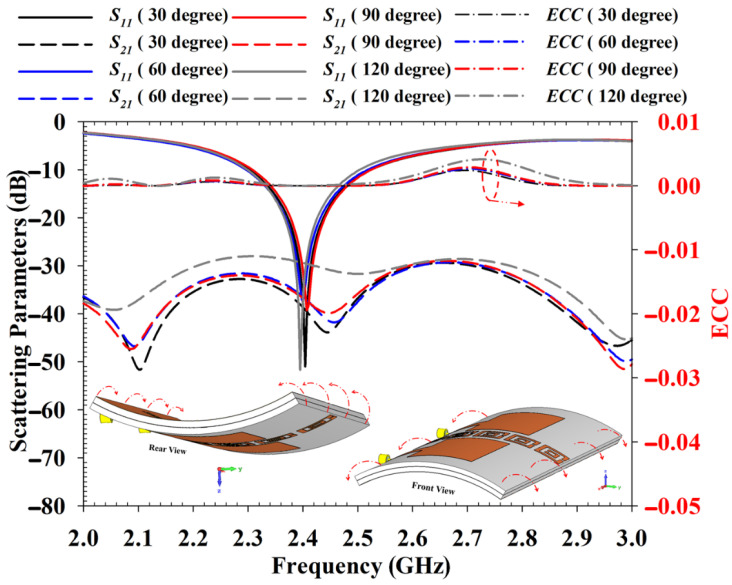
Bending analysis of the antenna at *y*-axis.

On the other hand, Figure 17 shows the bending effect when the antenna is bent along the *y* axis. The analysis indicates that the antenna *S*_11_ is shifted when the bending range is increased. It also can be noted that for 120 degrees, the *S*_21_ result was primarily affected where it was reduced to −30 dB. Therefore, it can be concluded that the bending along the *y* axis can increase the antenna’s mutual coupling. The information in this analysis is important as when the antenna is deployed in the human body, the bending along the *y* axis should be avoided or minimized. The analyzed ECC value is less than 0.01 dB regardless of bending conditions, which indicates the mutual coupling reduction is effective with EBG.

**Figure 18:**
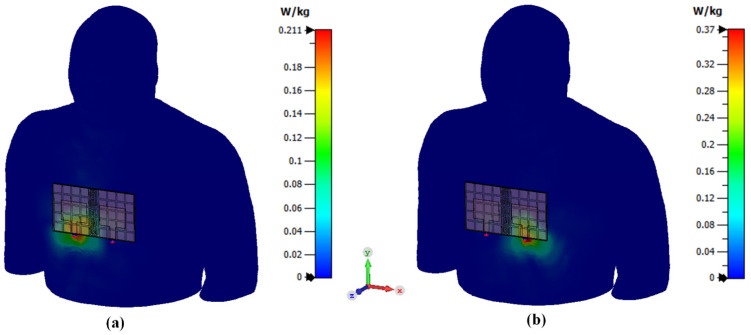
SAR analysis for 1g tissue. (**a**) antenna 1, (**b**) antenna 2.

**Figure 19:**
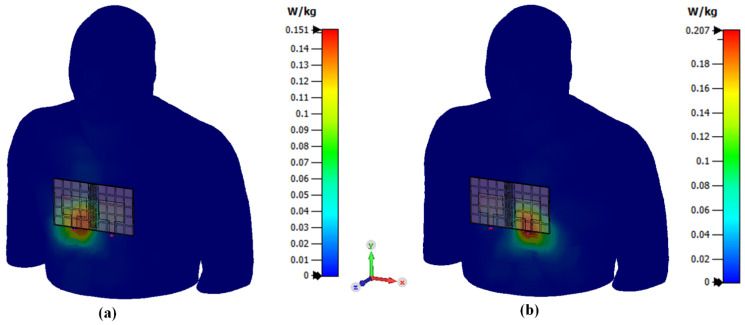
SAR analysis for 10g tissue. (**a**) antenna 1, (**b**) antenna 2.

Specific Absorption Rate (SAR) analysis was also conducted in addition to deformation analysis since the antenna could be used for wearable applications. The SAR results should be lower compared to the regulated SAR value. The regulated SAR value of 1.6 W/kg is taken over 1g of tissue that absorbs most EM energy and 2 W/kg is taken over 10 g of tissue that absorbs EM energy. The location of the antenna was selected to be on the chest of the body since the antenna size is considerably large with the MIMO method. Figure 18 and Figure 19 show that the peak rate of the SAR is 0.37 W/kg and 0.207 W/kg for 1 g and 10 g, respectively. The antenna at port 2 yields the maximum value for both SAR regulations. However, all SAR results are still below the regulated SAR value which is 1.6 W/kg taken over 1 g of tissue that absorbs most EM energy and 2 W/kg taken over 10 g of tissue that absorbs EM energy. These findings indicate that the proposed antenna is safe for on-body application. The use of RIS in this antenna also helps reduce the antenna’s back-lobe radiation pattern. Hence, properly implementation of the RIS structure helped reduce the SAR value.

A comparative analysis of the proposed high-performance textile antenna with previously investigated MIMO textile antenna is presented in Table 3 in terms of material used, operating frequency band, techniques used, antenna gain and isolation performance. The comparison shows that some works adopt materials that are difficult for fabrication, such as jeans as a substrate and copper sheet as the radiating element. None of the existing work has deployed two types of metamaterials in a single design to achieve different performance attributes as proposed in this work.

## 4. Conclusions

A metasurface-inspired textile MIMO antenna featuring both RIS and EBG surfaces was proposed and studied. The combined metasurface-inspired antenna prototype was fabricated using flexible polymer dielectric, viscose-wool felt as it enables easier fabrication with Shieldit Super^TM^. The RIS array mainly helped increase the gain and bandwidth of the patch antenna. On the other hand, the proposed strip line backed SRR EBG exhibited band stop properties in the desired frequency range, from 2.4 GHz to 2.5 GHz. Via-less EBG was chosen as the via could complicate the fabrication process with the textile antenna. The implementation of the RIS and EBG into the antenna design was proven experimentally, where they improved the antenna gain and bandwidth reducing the mutual coupling effects. Measurements showed *S*_11_ bandwidth from 2.16 GHz to 2.66 GHz for magnitude <−10 dB, with a peak gain of 5.8 dBi. The *S*_21_ was <−40 dB over the frequency ranges from 2.36 to 2.52 GHz. Apart from this, the proposed antenna exhibits acceptable results in terms of MEG and ECC. The bending analysis also showed that the antenna performance effect is very minimal when bent at the *x*-axes. The overall findings indicate that the proposed design has the potential to be applied for wearable applications as the SAR analysis also showed a good result where the SAR value was less than 1.6 W/kg taken over 1 g of tissue and less than 2 W/kg taken over 10 g of tissue that absorbing EM energy.

## Figures and Tables

**Table 1:** Parameters dimensions of the proposed antenna with RIS.

Para.	Value (mm)	Para.	Value (mm)
*W_s_*	110.0	*W_f_*	13.0
*L_s_*	106.0	*L_f_*	26.8
*W_p_*	60.0	*W_o_*	24.0
*L_p_*	40.0	*Y_o_*	5.5

**Table 2:** Parameter dimensions of the SRR backed with strip line EBG.

Para.	Value(mm)	Para.	Value(mm)
*a_e_*	21.0	*d_e_*	4.0
*g_e_*	2.0	*s_e_*	5.0
*b_e_*	18.0	*h_e_*	6.0
*c_e_*	8.0		

**Table 3:** Comparison of the proposed design with relevant previous work in the literature.

Reference	Material Used	Operating Frequency (GHz)	Metamaterial Structure/Technique	Antenna Gain (dBi)	Isolation (dB)	Remarks
[38]	Substrate: Viscose-wool felt Conductive sheet/element: Shieldit Super^TM^	2.4 & 5	Substrate integrated waveguide (SIW)	NA	20	The investigation was conducted on MIMO antenna performance, but no specific method was used to reduce the mutual coupling
[39]	Substrate: Jeans Conductive sheet/element: Copper sheet	2.74–12.0	8-shaped stub on a ground plane	6.9	26	Ultrawideband antenna design with 2 element MIMO Integration of copper sheet with jeans was not shared
[40]	Substrate: Jeans Conductive sheet/element: Copper sheet	3.5–8	Microstrip neutralization line	NA	32	Ultrawideband antenna design with 2 element MIMO Integration of copper sheet with jeans was not shared
[19]	Substrate: Standard felt Conductive sheet/element: Cotton fabric	1.1–8.6	-	7.5	40	The use of cotton fabric as the patch in the antenna design is not practical. While no specific mutual coupling reduction technique was used to achieve high isolation.
[28]	Substrate: Jeans Conductive sheet/element: Copper sheet	1.5–3.8 4.2–6.2	Meanderline	2–5	25–33	A Dual-band antenna was designed with 4 element MIMO Integration of copper sheet with jeans was not shared
This work	Substrate: Viscose-wool felt Conductive sheet/element: Shieldit Super^TM^	2.16–2.66	RIS EBG	5.8	40	RIS was used to miniaturize the antenna, increase the antenna gain (+1.29 dBi) and bandwidth. Mutual coupling reduction in MIMO antenna

## Data Availability

The study did not report any data.
